# Assessment of BG-Pro (Biogent AG) and Silver Bullet 2.1 (Lumin8) UV-Light Traps Efficiency for Surveillance of Malaria Vectors in Western Kenya

**DOI:** 10.3390/insects16070739

**Published:** 2025-07-19

**Authors:** Billy L. Amugune, Richard Tamre, Dylan Mogaka, Oscar Mbare, Tullu Bukhari, Ulrike Fillinger, Margaret M. Njoroge

**Affiliations:** Global Health Thematic Research Programme, International Centre of Insect Physiology and Ecology (ICIPE), Nairobi 00100, Kenya

**Keywords:** *Anopheles*, mosquitoes, sub-Saharan Africa, trap design, power bank

## Abstract

The Centers for Diseases Control (CDC) light trap is widely used for malaria vector surveillance, but its acquisition logistics pose challenges in Africa. Evaluating new traps can improve surveillance tools. This study compared the efficiency of the BG-Pro UV and Silver Bullet 2.1 UV (SB 2.1 UV) against the UV LED CDC trap in western Kenya’s rice irrigation area. The traps were tested indoors in eight houses over 64 nights. Light properties and fan speed were analyzed using spectrometry and an anemometer. The BG-Pro UV trap performed better than the UV LED CDC trap for *An. gambiae s.l.* (RR 2.0, 95% CI 0.9–3.9) and *An. funestus s.l.* (RR 3.5, 95% CI 1.9–6.4). The SB 2.1 UV trap was more effective in capturing *An. gambiae s.l.* (RR 4.3, 95% CI 2.5–7.3) and *An. funestus s.l.* (RR 7.1, 95% CI 3.9–13.1), and also caught three times more *Culex* spp. (RR 2.7, 95% CI 1.2–6.0). SB 2.1 UV had the highest downstream force, and all traps emitting UV-A light had consistent wavelengths. Overall, the BG-Pro and SB 2.1 traps’ trapping efficiency was three to six times more than the CDC trap, making them promising surveillance tools, particularly in low-density malaria settings.

## Simple Summary

Light traps are often used in malaria research to catch mosquitoes; this helps scientists keep track of the number of mosquitoes that spread malaria in a particular area and check if control measures are working. One trap, the Centers for Diseases Control (CDC) light trap, is commonly used in Africa where malaria is common. However, obtaining this trap can be challenging due to logistical difficulties. So, it is useful to try other traps that might work equally well or better to add to the traps used for mosquito surveillance. In this study, two new UV-light traps—the BG-Pro and the Silver Bullet 2.1—were tested to see how well they catch female *Anopheles* mosquitoes, which spread malaria. Both new traps caught more mosquitoes than the CDC trap. This means they could be useful tools for future mosquito research.

## 1. Introduction

Vector surveillance is vital for management programs of vector-borne diseases, as it enables the assessment of the impact of an intervention strategy for better planning, resource mobilization, testing, improvement, and the deployment of effective control tools [1]. Several trapping systems are available for mosquito surveillance. Most of these are light traps targeting *Anopheles* mosquitoes, and they are widely used due to their ease of handling, non-invasive nature of operation, and location versatility compared to baited systems [2]. To date, the CDC light trap fitted with incandescent bulbs remains the most widely used trap for the surveillance of malaria vectors [3,4,5]. Incandescent light bulbs produce heat as they radiate most of their energy in the infrared light region, and the heat source might add to the attraction of the trap, especially when there is no other warm-blooded host nearby [6,7]. However, light-emitting diodes (LEDs) are more energy-efficient sources of light, which is especially beneficial when traps can only be operated on batteries [8,9]. Ultraviolet light-emitting-diodes (UV LEDs) are increasingly being adopted and tested for the surveillance of mosquito populations, as they emit light that is in the visible spectrum to mosquitoes compared to the white LEDs, which produce most of their energy in the light spectrum, which is not visible to mosquitoes; hence, this adds little additional attraction to a trap [10,11].

The CDC light traps are standard traps for the surveillance of adult *Anopheles* mosquitoes indoors and outdoors [4,7,12]. However, obtaining these traps for use in sub-Saharan Africa involves logistical hurdles that have recently been further complicated by the closure of a major distributor [13]. The evaluation of other trap models in comparison to the CDC light traps is important to expand the options available to programs and research institutes.

The BG-Pro UV-light trap manufactured by Biogents AG, Germany, provides a potential addition for malaria vector surveillance in sub-Saharan Africa. The BG-Pro UV trap is uniquely built with parts that provide options to dismantle the trap for ease of transportation [14]. The trap was initially developed for the monitoring of *Aedes* mosquitoes; however, with the rising need for additional malaria-vector surveillance tools in Africa, such tools can be repurposed to enhance the monitoring of emerging malaria mosquitoes [15,16,17]. The Silver Bullet 2.1 UV trap (Lumin8, Germiston, South Africa) is another novel trap that has been shown to have great potential in sampling malaria vectors in Africa [7]. This study aimed to evaluate the efficiency of the BG-Pro UV in trapping malaria vectors compared to the novel Silver Bullet 2.1 UV and the wildly used UV LED CDC light trap. All traps were operated with LEDs in the UV-light range according to the manufacturer settings.

## 2. Materials and Methods

### 2.1. Study Site

The field studies were conducted between July and September 2023 in Kigoche village located within the Muhoroni sub-county in Kisumu county (Longitude 34.912376, Latitude −0.151164). The area is hyperendemic for malaria and rice irrigation farming, although considered an important malaria risk factor, is the major economic activity [18,19]. This study selected eight similar double-room houses, each measuring approximately 15 m^2^, featuring traditional construction typical of the area—smooth mud walls and corrugated iron sheet roofs [20]. Eligible households included at least one child and two adults. The eaves under the roof of all the selected houses were open and the houses were at least 300 m apart. 

### 2.2. Light Traps

#### 2.2.1. BG-Pro UV-Light Trap 

**Figure 1:**
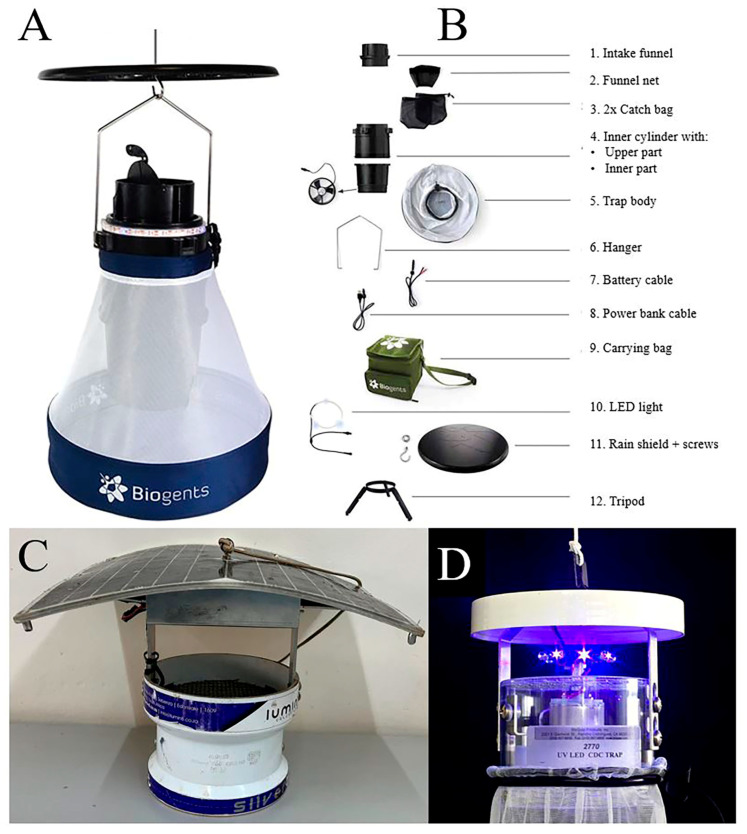
(**A**) BG-Pro UV trap—fully assembled; (**B**) all trap components of BG-Pro UV fully disassembled; (**C**) Silver Bullet 2.1 UV-light trap; and (**D**) UV LED CDC light trap.

The BG-Pro UV-light trap (Biogents AG, Regensburg, Germany) is a cone-shaped fabric trap (Figure 1A) equipped with several accessories (Figure 1B, 1–12). The trap could be powered by a 6-volt battery or a 5-volt lightweight rechargeable power bank that runs a three-blade fan, which generates a bidirectional airflow that draws mosquitoes into the trap’s catch bag [14]. The trap is fitted with a black catch bag (Figure 1B, 3) located above the fan to collect trapped mosquitoes. The catch bag is made of a downward-facing funnel net notably positioned above the fan to prevent the catches from escaping once trapped. In addition, the positioning of the catch bag above the trap prevents damage to the trapped mosquitoes. The LED strip (Figure 1B, 10) fitted around the intake funnel and just below the mosquito entry point emitted UV-light to attract mosquitoes towards the trap. The trap’s white body houses the trap components, including the power bank. The UV LED light ring with LED lights in the form of a strip is optimally mounted on top of the trap cylinder to lure mosquitoes towards it (Figure 1A). In the present study, both power options were tested to assess if the power source might affect the operation of the trap and consequently the trapping efficacy.

#### 2.2.2. Silver Bullet 2.1 UV

A comprehensive description of the Silver Bullet 2.1 light trap is provided in a previous study in 2023 by Mbare and colleagues [7]. Briefly, the trap manufactured by Lumin8, Germiston, South Africa, has an inbuilt rechargeable lithium-ion battery pack that can either be solar recharged through a 12 W polycrystalline solar panel fitted on top of the trap or recharged through mains power (Figure 1C). It has three LED clusters located above the mosquito entry point that offer five differently coloured lights [7]. The fan of the SB 2.1 UV is situated above the catch bag. For this study, the UV LED setting was selected, as it was previously found to be attractive to *Anopheles* mosquitoes [7].

#### 2.2.3. UV LED CDC Light Trap 

The UV LED CDC trap has eight ultraviolet LED platform diode options [21,22]. These diodes are located above the designated mosquito entry point (Figure 1D). This trap was powered with a 6-volt external battery. The white catch bag in the CDC light trap was fitted below the fan. 

### 2.3. Light Properties and Fan Speed

To ensure consistent trapping efficiency across all UV-light traps, we measured and compared their light wavelengths and irradiance levels using spectrophotometry. This controlled for potential variations in emitted light waves and fan speeds that could affect trap performance [23]. Measurements were taken first at the source by placing the trap in front of the spectrophotometer probe and then at a 1 m distance by moving the trap but keeping the probe stationary. Readings were taken in triplicate and the means were used to plot light spectra (Figure S1). Fan speed was measured using a Kestrel 1000 wind meter (Kestrel Meters, Boothwyn, PA, USA) calibrated in miles per hour (mph); data from three independent measurements were averaged and used to estimate the gravitational force (*g*-force) of each trap relative to the fan radius using the equation (Relative Centrifugal Force = (Revolution Per Minute)^2^ × 1.118 × 10^5^ × r) to quantify the downstream force created by the fan revolution [24].

### 2.4. Experimental Design

The indoor trapping efficacy of four light traps—BG-Pro UV operated using a power bank, BG-Pro UV operated using a 6 V battery, SB 2.1 UV operated by an inbuilt rechargeable power bank, and UV LED CDC operated using a 6 V battery—were compared in this study. The eight experimental houses selected for this study were divided into two blocks, each with four houses. On any experimental night, the traps were set in the four houses belonging to each group. The traps were rotated on each subsequent night, ensuring that every trap was placed in each of the four houses over four consecutive nights. Thereafter, the nightly trap rotation was transferred to the other group of houses and the experiments were conducted over the next four consecutive nights. The entire experiment was conducted over 16 nights, giving a total of 64 trap nights for the four traps (16 nights × 4 traps/night). The traps were set indoors at approximately 1 m from the ground at the foot end of the bed where the adult household head slept. No additional artificial bait or lure other than the individuals on the bed was used. Long-lasting insecticidal nets were provided for all sleeping places in every house and residents were requested to use them during trap nights. Traps were run all night long from 1830 h to 0630 h. This study was conducted to completion during the dry season in an area where rice paddies were dry and not under cultivation.

### 2.5. Mosquito Identification

Catch bags from all traps were transferred in the mornings to a field-based laboratory where they were placed in a freezer for 30 minutes to kill the mosquitoes. Afterwards, mosquitoes were removed from the catch bag and morphologically identified using reference keys [25]. Anophelinae were differentiated up to the species level while Culicinae identification was performed only up to genus level. Molecular assays were performed on the *Anopheles gambiae sensu lato* and *Anopheles funestus sensu lato* complexes. DNA preparation was performed following the ammonium acetate protocol described in Amugune et al. 2022 [26], and PCR amplification was performed as described in Scott et al. 1993 [27].

### 2.6. Statistical Analysis

Generalized estimating equations (GEEs), in IBM SPSS (version 25), were used to analyze the data. Mosquito counts were analyzed by fitting a negative binomial distribution with a log link function. The trap type was included in the model as a fixed factor; the battery powered BG-Pro and one powered by a power bank were considered as separate traps. The experimental night was included in the model as a repeated measure after the analysis of variance of repeated measures indicated no significant variation in catches within the houses. Rate ratios (RRs) and estimated model means, with their associated confidence intervals (CIs), were obtained as an outcome of the model. Analyses were performed separately for counts of *Anopheles arabiensis*, *An. funestus s.s.*, and *Culex* spp. whilst the number of the other *Anopheles* species (*An. coustani* and *An. pharoensis*) and Culicinae sub-family species (*Mansonia* spp., *Aedes* spp., *Coquilletidia* spp.) trapped were too low for further statistical analysis.

### 2.7. Ethical Considerations

This study was conducted following the guidelines of the Declaration of Helsinki and with ethical approval from KEMRI-SERU (NON-KEMRI PROTOCOL NO. 4520). An informed consenting procedure was conducted for all the households that participated in this study prior to the commencement of any activities. 

## 3. Results

### 3.1. Trapping Efficacy

A total of 4089 mosquitoes were captured during 64 trap nights. Most of the trapped mosquitoes (90.7%; n = 3708) were females. Of all the females, nearly a quarter (24%, n = 889) were from the Anophelinae sub-family, whilst the majority (76%; n = 2819) were from the Culicinae sub-family. Of the female Anophelinae, 77.8% (n = 691) were *Anopheles arabiensis*, 20.7% (n = 184) were *An. funestus s.s.*, 1.3% (n = 12) were *An. coustani*, and 0.2% (n = 2) were *An. pharoensis*. No *An. gambiae s.s.* nor other sibling species of the *An. funestus* complex were identified through molecular analysis. Of the female Culicinae, 95.2% (n = 2683) belonged to the genus *Culex* and 4.7% (n = 132) to the genus *Mansonia*. Only two specimens (0.07%) were collected for both *Coquilletidia* and *Aedes*. Of the trapped male mosquitoes, (n = 250), the majority were *An. arabiensis* (41.2%, n = 157) followed by *An. funestus* (24.4%, n = 93), with the remainder being *Culex* (33.3%, n = 127) and *Mansonia* species (1.0%, n = 4).

Using either a 5 V lightweight power bank or 6 V battery did not significantly affect the trapping efficacy of the BG-Pro UV trap for any species (Table 1).

Compared to the UV LED CDC trap, the BG-Pro UV trap, powered by either a battery or a power bank, caught twice as many *An. arabiensis*. The battery-powered BG-Pro UV performed almost four times better than the UV LED CDC trap, while the power bank-powered BG-Pro UV trap performed five times better than the UV LED CDC trap in catching *An. funestus s.s.* (Table 1). However, the efficacy of both the BG-Pro UV traps and UV LED CDC traps in trapping *Culex* was similar (Table 1).

The SB 2.1 UV-light trap showed the best performance from all the four tested traps in catching female *Anopheles* and *Culex*. It trapped approximately four times more female *An. arabiensis*, seven times more female *An. funestus s.s.*, and approximately three times as many *Culex* compared to the UV LED CDC light trap (Table 1). Compared to the BG-Pro UV, powered by either a battery or power bank, the SB 2.1 UV-light trap caught approximately two times as many *An. arabiensis* and *An. funestus s.s.*, and approximately three times as many *Culex* spp. (Table 1).

### 3.2. Comparison of Fan Speed and Light Properties

The three traps were fitted with fans of different sizes and, combined with their power sources, generated varying fan speeds (Table 2). The CDC light trap generated the lowest gravitational force, whilst the SB 2.1 trap generated nearly double the *g*-force than the UV LED CDC trap (Table 2).

All three light sources used in the three different traps generated light in the UV-A spectrum. The UV LED CDC emitted a peak at 388 nm, whilst the BG-Pro had its peak at 365 nm, and the SB 2.1 UV at 400 nm. The light emitted from the traps was only detectable at the source at a very close proximity (0 m).

## 4. Discussion

This study evaluated the efficiency of the BG-Pro UV-light trap in catching mosquitoes in an indoor environment. Comparisons were made with the common standard UV LED CDC light trap and another novel trap, the Silver Bullet 2.1. Based on the mean number of mosquitoes collected, the BG-Pro UV-light trap, powered by either a power bank or a battery, outperformed the UV LED CDC light trap in collecting the major malaria vectors in the field site, which warrants its use as a surveillance tool for these predominant malaria vectors in sub-Saharan Africa. 

The advantage of the BG-Pro UV over other traps is its lightweight and the collapsible body which can be packed into a small bag, which is easy to carry. Thus, compared to the CDC light trap, the BG-Pro presents a logistical improvement, especially when lightweight power banks charged with USB-compatible power sources can be used in place of heavy batteries used in CDC traps [14]. However, despite these advantages, the BG-Pro UV-light trap was out-competed by the recently profiled SB 2.1 UV [7]. The Silver Bullet 2.1 trap caught double the number of malaria mosquitoes than the BG-Pro UV and four times as many as the UV LED CDC. All traps were fitted with LED lights, and the spectrophotometer results confirmed that all traps emitted a similar light quality in the UV-A spectrum; thus, this is unlikely to be responsible for the differential trapping efficacy. Important is the finding that none of the traps’ lights were visible 1 meter or more away from the trap, highlighting that the light emitted from these traps does not serve as long-range attractant cues to mosquitoes [28]. Other cues, such as body odor and CO_2_, are hence essential for longer ranges to lure the mosquitoes towards the trap [29]. 

The *g*-force of the traps can affect their efficiency of sucking insects into the collection chambers [30]. The BG-Pro trap had a greater *g*-force than the CDC trap, and the SB trap generated the strongest force, which is the most likely explanation for the differences in trapping efficiency. In terms of costs, the BG-Pro came at the lowest price in 2023. At the time, the cost for one SB 2.1 UV trap was USD 205, USD 190 for the UV LED CDC trap and, USD 103.93 for the BG-Pro. In addition, from our observation, the delivery time for the BG-Pro was significantly shorter compared to that for the other traps.

However, the BG-Pro UV trap presented a few challenges for its use in the field. One of the challenges was the frequent breakdown of the LED light band, which required constant repairs, increasing operational costs when deployed for vector surveillance in the field. The catch bag was torn easily and quickly after consistent use, as the material is very fragile and not selected for wear and tear. Lastly, the tripod stands which are used to shape the trap are made of plastic material that breaks easily (Figure 1B, 12). All these factors present durability challenges for trap use but could be easily addressed by the manufacturer. 

This study was conducted exclusively indoors during the dry season when vector densities were low, with traps installed in houses adjacent to uncultivated fields. Data collection occurred over a limited time period. Given that *Anopheles* populations exhibit strong seasonal and cropping cycle-dependent fluctuations, extended spatiotemporal studies are needed to more accurately assess the traps’ capture efficiencies. 

## 5. Conclusions

The BG-Pro trap demonstrated the potential for sampling indoor malaria vectors under field conditions. The ease of the trap usage in combination with its costs would be advantageous in scalability aspects and also usage in resource-poor settings. Although this is so, it would be prudent to conduct additional studies to assess its performance in an outdoor environment, in different seasons, in areas with varied vector densities, and in comparison with other traps. Moreover, it is necessary to ensure that its parts are made of durable material to reduce the frequency of replacement and repair that could affect logistics and rational costs.

## Figures and Tables

**Table 1:** Compilation of pairwise statistical outputs comparing relative risk of trapping female *Anopheles arabiensis*, *An. funestus*, and *Culex* spp. by 4 trap set ups (UV LED CDC, Silver Bullet 2.1 UV, BG-Pro UV battery-powered, and BG-Pro UV power bank-powered).

Trap	Mean (95% CI)	RR (95% CI)	*p*-Value	RR (95% CI)	*p*-Value	RR (95%CI)	*p*-Value
*An. arabiensis*							
UV LED CDC	5.1(3.5–7.6)	1	-				
Silver Bullet 2.1 UV	21.9 (15.1–31.8)	4.3 (2.5–7.3)	<0.001	1	-		
BG-Pro UV (Battery)	10.0 (5.6–17.8)	2.0 (0.9–3.9)	0.06	0.5 (0.2–1.0)	0.043	1	
BG-Pro UV (Power bank)	11.0 (5.9–20.5)	2.1 (1.3–3.6)	0.005	0.5 (0.2–1.1)	0.066	1.1 (0.6–2.0)	0.760
*An. funestus s.s.*							
UV LED CDC	0.9 (0.6–1.4)	1	-				
Silver Bullet 2.1 UV	6.3 (4.0–9.8)	7.1 (3.9–13.1)	<0.001	1	-		
BG-Pro UV (Battery)	3.1 (1.7–5.6)	3.5 (1.9–6.4)	<0.001	0.5 (0.2–1.1)	0.076	1	
BG-Pro UV (Power bank)	4.4 (2.5–7.8)	5.0 (2.7–9.3)	<0.001	0.7 (0.4–1.3)	0.276	1.4 (0.7–3.0)	0.348
*Culex* spp.							
UV LED CDC	32.9 (18.5–58.7)	1	-				
Silver Bullet 2.1 UV	88.9 (42.3–187.0)	2.7 (1.2–6.0)	0.014	1	-		
BG-Pro UV (Battery)	25.0 (15.4–40.7)	0.8 (0.6–1.3)	0.292	0.3 (0.1–0.6)	0.001	1	
BG-Pro UV (Power bank)	26.8 (15.8–45.4)	0.8 (0.3–1.9)	0.640	0.3 (0.1–0.8)	0.011	1.0 (0.6–2.1)	0.842

**Table 2:** Exploration of light trap fan speed of 4 traps (UV LED CDC, BG-Pro UV powered by power bank, BG-Pro UV powered by battery, and Silver Bullet 2.1 UV) in relation to the fan radius to estimate the downdraft *g*-force exerted for mosquito trapping.

Trap	Fan Radius	Fan Speed	G-Force
UV LED CDC	4 cm	6.2 mph	20 × g
BG-Pro UV (power bank)	6 cm	8 mph	22 × g
BG-Pro UV (battery)	6 cm	10.1 mph	35 × g
Silver Bullet 2.1 UV	4.5 cm	9.0 mph	37 × g

## Data Availability

The raw data supporting the conclusions of this article will be made available by the authors on request.

## Supplementary Materials

The following supporting information can be downloaded at: https://www.mdpi.com/article/10.3390/insects16070739/s1, Figure S1. Comparison of light properties (light wavelength in relation to absolute intensity of light) of UV platform of all traps.
